# Electrical circuit model of spatiotemporal trade dynamics: Foundations and derivation of the gravity model

**DOI:** 10.1371/journal.pone.0326102

**Published:** 2025-06-17

**Authors:** P.A. Robinson, Alexander McInnes, Najmeh Sajedianfard, Mark Melatos, James A. Henderson

**Affiliations:** 1 School of Physics, University of Sydney, Sydney, New South Wales, Australia; 2 Department of Economics, Faculty of Administrative Sciences and Economics, University of Isfahan, Isfahan, Iran; 3 School of Economics, University of Sydney, Sydney, New South Wales, Australia; Aalto University, FINLAND

## Abstract

A model of time-dependent trade of goods between spatial locations is formulated via an electric circuit analogy, in which goods are analogous to charge and price to voltage, while producers and consumers are represented by sources and sinks of goods flow, which is represented by current, located at the nodes of a trade network. The core ansatz is that the flow of goods along each network link is driven by the voltage difference across that link, opposed by resistance that represents trade friction. Market prices are then determined indirectly by internal balances of flows, subject to external constraints on supply and demand. The model yields multiple outcomes that support its validity and applicability, including price setting via emergent balance of supply and demand, price fluctuations, traditional and generalized elasticities, network structure-flow relations, competition between producers, and substitution between suppliers, between consumers, and/or between trade links. All these results prove to be consistent with observed features of trade dynamics, thereby supporting the validity of the model. The new model is then used to derive the widely used gravity model of international trade from a mechanistic basis, yielding exponents consistent with published data and leading naturally to core-periphery structure, as observed in real trade networks. The analysis also implies that trade flows self-organize to minimize trade friction in the system as a whole, an emergent global outcome from the purely local dynamics of the populations of producers, consumers, and traders. Possible generalizations and further applications are outlined, including incorporation of asymmetry and capacity limits of trade links, constraints on supply and demand, behavioral responses, and coupling to models of investment strategies.

## 1 Introduction

Commerce and trade have been central to economies for thousands of years and have increased steeply in the last century as globalization and falling transport costs have enabled increasing specialization and resulting economies of scale. Many aspects of trade are dynamic in the sense that they are time-dependent; these include supply and demand changes, seasonal factors, transport, and the entry of firms into, and exit from, export markets [1,2].

The theoretical economics literature on international trade has traditionally employed static equilibrium models that hold factors of production and technology fixed [3] or undertake comparative static analyses of the impacts of some trade policy change or a change to model parameters [4]. Econometric models, on the other hand, especially the gravity model of international trade, have been used extensively to empirically address many individual facets of the above issues [5–13].

Of the economic models that explicitly model trade dynamics – and most still do not – the focus tends to be on measuring the gains from trade over time [14], the relationship between trade and economic growth [15], the role of uncertainty in trade policy choice [16], the role of trade policy uncertainty itself [17] and changes to the so-called extensive and intensive margins of trade, which relate to changes in new and existing trading relationships, respectively [18]. Nevertheless, most trade models omit dynamics and often focus only on mean trade volumes between countries, for example; similarly, few consider geographic factors [5,6]. Some key questions thus center on how time-varying prices and trade flows are determined by supply and demand changes, how much trade flows between locations, how transport capacity affects trade, the effects of stockpiles and inertia in trade networks, distance dependences of trade volumes, the elasticities of supply and demand, and how prices change in response to external factors. While economic and econometric models have treated specific facets of the above issues no model has combined them, especially spatial aspects, in as integrated a way as proposed here. It is worth stressing that, as a result of these limitations, the role of international trade dynamics and the relationships between trade network structure and trade flows are still not well understood.

The aim of the present work is to provide an initial semiquantitative model that can answer many of the above questions, probe their interrelationships, and provide a foundation for further generalizations. To keep the scope manageable, we do not attempt to include all possible features and we exclude wider issues such as investment decisions, futures, and options. Likewise, because of the need to develop the mathematical basis of the model, the detailed analytic work in the present paper is mainly aimed at econophysicists who are interested in mechanistic modeling; however, the applications are economic.

Rather than making a series of traditional economic assumptions, our model is based on an electric-circuit analogy, in which a product is produced, consumed, and marketed at nodes of a network that model physical locations of supply, demand, and trade. (The case of multiple products with different characteristics is reserved for future work in order to concentrate on the trade network here.) The central ansatz is that flows of goods from producers to consumers are modeled as currents that are driven by voltage differences across network links, with voltage being identified with the negative of price because prices rise in moving from producer to consumer; voltage differences between nodes drive flows of goods from high voltage (low price) to low voltage (high price). Transport, tariffs, and taxes are incorporated via electrical resistance, which measures trade friction; electrical inductance models flow persistence or inertia due to goods in transit, contractual obligations, and other effects that oppose sudden changes in trade flows; while storage of goods can be included via electrical capacitance. In some respects, the model recalls aspects of the classical MONIAC model, in which conserved flows of money through an economy were modeled by pressure-driven flows of water through an electromechanical apparatus of pipes and valves [19]. The use of electrical analogies preserves the ability to obtain physical insights but is far more flexible in general because these can more easily include nonlinear effects and their parameterization and solution can be done via much more powerful and widely available electrical circuit software.

We develop and apply our core model in stages in the subsequent sections. In Section 2 we explain our general stepwise modeling strategy and then formulate the model for a single locality, with supply and demand (producer and consumer) nodes. The model is then extended to include market nodes that are linked to other locations to form a spatially extended trade network in Section 3, and measures of trade flows, elasticities, and fluctuations are established. Then in Sections 4 and 5 we apply the model to analyze a range of applications, verify that its central predictions are qualitatively in accord with known trade phenomena, and identify areas requiring further refinement. We apply it to derive and generalize the gravity model of trade and show that its predictions are consistent with prior data. Additionally, we undertake an initial tentative application to high frequency trading of stocks and commodities to explore the dynamics further and indicate promising avenues for further model development. Section 6 summarizes the main results and outlines some of the many extensions that could be based on the present foundation.

## 2 Single-location model

In this section we first explain our general, stepwise strategy in which our new model is to be developed and tested in successive stages, with the first stages presented in this paper. This approach is common in physical modeling but is less familiar in economics, where a few widely-known models predominate.

In the following subsections, we formulate the model for a single spatial location, which could represent that of an individual producer or consumer, a whole province or country, or even a group of countries, depending on the spatial resolution desired. We then connect locations into a network of production (supply), consumption (demand), and trade in Section 3. We first consider a purely resistive model that models costs associated with production and consumption, prior to generalizing to an impedance model to allow for dynamical effects in the system. An advantage of this formulation is that thoroughly tested engineering software for solving electrical circuits is widely available and can be adapted for use both in the present context and in the suggested future generalizations [20].

The mathematical development of the model requires some familiarity with alternating current electric circuits, but the qualitative features and outcomes should be accessible by an interdisciplinary audience, for whom some additional explanation is provided at relevant points, while texts such as [21,22] provide full details.

### 2.1 Modeling strategy

Perhaps the most important point to note is that the present mechanistic model starts from a different basis from traditional economic models of trade. Hence, it does not begin by incorporating traditional assumptions such as balance of supply and demand; rather such features emerge as consequences of the core ansatz that flows of goods are driven by voltage differences. Indeed, in common with physics-based modeling of other systems, our aim is to reproduce as many outcomes as possible from the fewest assumptions. The utility of the model is then assessed by the extent to which it reproduces observed economic and trade phenomena, as would be the case when modeling more commonly studied physical systems.

In this first version of the model, which integrates the most salient features of trade systems, we assume linear dynamics to keep the work manageable, but pointers to nonlinear and other extensions are given in many places. In this paper we verify that the behavior of the model is qualitatively consistent with economic behaviors such as the balance between supply and demand, elasticities, and substitution, starting with a resistive single-node model. The results are then generalized to include trade between multiple nodes and dynamic effects, thereby allowing the derivation of the gravity model of trade and its verification against prior data. Directions for further generalization and verification in future stages of model development are indicated at the appropriate points.

### 2.2 Single-location resistance model

**Fig 1:**
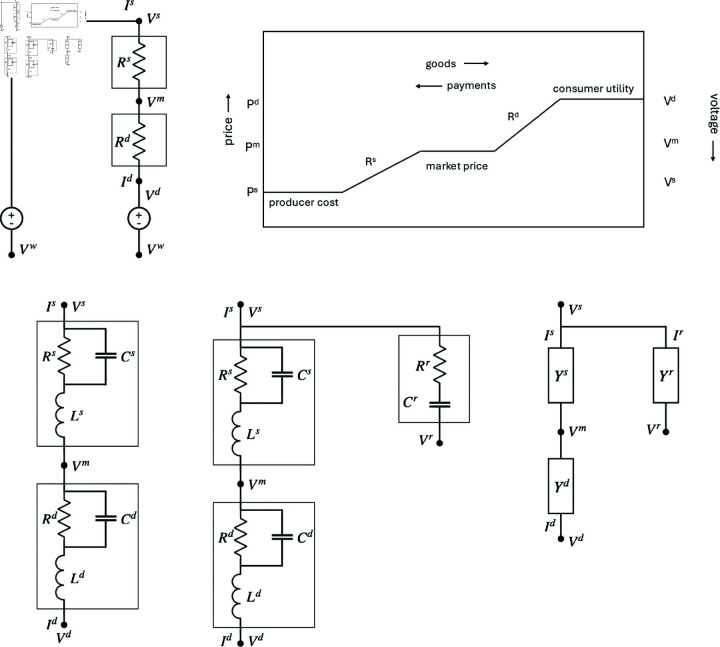
Single-location electric circuit models showing supply (s) and demand (d) sectors with correspondingly superscripted voltages V (i.e., negative prices P), currents I (flows of goods), resistances R, capacitances C, inductances L, and admittances Y. (top left) Pure resistance model showing current flow from the supply voltage $V^{s}$ through resistance *R*^*s*^ to the market voltage $V^{m}$. After this it flows through the demand sector to voltage $V^{d}$. All voltages are measured relative to a large negative reference value $V^{w}$ and voltage sources maintain the values $V^{s}$ and $V^{d}$. (top right) Schematic of price and voltage changes between producer, market, and consumer, also showing the direction of flow of goods and payments. (lower left) Model augmented with internal inductances in each sector. Here the links containing voltage sources are omitted for compactness. (lower middle) Further generalization to include the voltage $V^{r}$ that embodies a reserve price, with corresponding storage of electric charge (goods) in capacitor *C*^*r*^. (lower right) Same as previous frame but shown in terms of admittances *Y*, discussed in Sec. 2.4.

We begin by supposing that there is no trade (i.e., autarky) and what is produced in one location is consumed there—i.e., that it flows directly from the production (supply) node at that location to the consumption node, where it satisfies demand, with the flow driven by payment of money. We also assume that there is a single homogeneous good that is produced and consumed. The demand will depend on the unit price that the local consumer is willing to pay (i.e., their marginal utility), as well as external factors such as variations in the rates of supply (e.g., different crop yields) and demand (e.g., seasonal changes). We model this via the ohmic resistance circuit shown in Fig 1 at the top left, where the current *I*^*s*^ represents the rate of supply of goods (i.e., goods produced per unit time). Goods enter the production process at a pre-production voltage $V^{s}$ and exit at a market voltage $V^{m}$, with

$$V^{m}=V^{s}-I^{s}R^{s}.$$ (1)

Voltages here are the negative of prices and thus decrease in moving from producer to consumer due to internal resistance (*R*^*i*^; i=s,d) in the production process; the meaning of this resistance will become apparent shortly. (Note that superscripts here indicate different sectors of the model and are not exponents; subscripts are reserved to denote different locations below.) Goods enter the consumption (demand) sector at the market voltage $V^{m}$ and exit at the post-consumption voltage

$$V^{d}=V^{m}-R^{d}I^{d}.$$ (2)

Since only voltage differences are relevant to driving currents, all voltages can be measured relative to a large negative reference voltage $V^{w}$, which can be arbitrary or can be used to represent the overall unit cost that is written off when generating post-consumption waste; voltage sources maintain the values $V^{s}$ and $V^{d}$ relative to $V^{w}$.

We interpret this local model in the following way: (i) Because the zero of voltage is arbitrary, it can be chosen so that the unit price *P* of goods can be taken to be the negative of voltage, P=−V so flow down a voltage gradient is equivalent to flow up a price gradient (equivalently, payments flow down the price gradient), as seen at the top right of Fig 1; thus voltages are normally negative here, which poses no difficulty because only voltage differences drive currents. Positive voltages (i.e., negative prices) might occur under conditions of glut, in which the producer would pay to have the product removed. (ii) The market price $P^{m}=-V^{m}$ is the price at which the consumer purchases the good. (iii) The final price $P^{d}=-V^{d}$ is what the consumer is willing to pay (i.e., their marginal utility), including the costs of preparing the good for use after purchase, which costs can be set to zero by putting *R*^*d*^ = 0. The voltage $V^{d}$ may be affected by a host of demand-side factors that we do not model here, but would include dependence of demand on the actual quantities of goods purchased—i.e., diminishing marginal utility at large flows (satiation) and budgetary constraints—which would require nonlinear circuit elements to model. (iv) Likewise, $P^{s}=-V^{s}$ represents the unit cost of inputs to the producer (i.e., the marginal cost of production), which is influenced by supply-side factors and gives the minimum price at which the producer can sell. As in (iii), inclusion of supply-side factors such as economies of scale would involve nonlinear circuit elements. (v) The voltage drop $I^{s}R^{s}$ is the sum of unit producer profit and the unit costs of production. (vi) The voltage $V^{m}$ is the (negative) unit price paid for the good given $V^{s}$ and $V^{d}$; in this simple case, supply *I*^*s*^ equals demand *I*^*d*^, so goods are conserved and solving Eqs (1) and (2) yields the explicit expressions

$$I^{s}=I^{d}=\frac{V^{s}-V^{d}}{R^{s}+R^{d}},$$ (3)

$$V^{m}=\frac{V^{s}R^{d}+V^{d}R^{s}}{R^{s}+R^{d}},$$ (4)

with $V^{d}=V^{m}$ if *R*^*d*^ = 0. Equation (3) shows that the flow is proportional to the price difference $V^{s}-V^{d}$ between producer and consumer that drives it, and inversely proportional to the total resistance $R^{s}+R^{d}$ between them. Equation (4) correctly implies that the market voltage always lies between the supply and demand voltages. The resulting relationship between *I*^*s*^ and $V^{m}$ gives a point on the demand curve, which is thus derived as a finding from the model, instead of being separately specified at the outset, as in many studies. It is also worth noting that money can be considered to flow in the reverse direction to goods, although we do not track money separately in the present work; nor do we consider issues such as investment decisions, borrowing, hedging, and capital accumulation. Multiple goods could be treated by including an analogous set of equations for each good; however, a model of consumer preferences and budget would then need to be added.

Another way of viewing this model is in terms of conductance *G*, which is the reciprocal of resistance; conductance thus measures the ease with which current can pass. In this case,

$$I^{s}=G^{s}(V^{s}-V^{m}),$$ (5)

$$I^{d}=G^{d}(V^{m}-V^{d}),$$ (6)

where $G^{s}=1/R^{s}$ and $G^{d}=1/R^{d}$. An increase in supply at a fixed price difference thus corresponds to an increase in the supply conductance *G*^*s*^, which is proportional to the price elasticity of supply (we explain this point further in Sec. 3.6). Economies of scale might increase *G*^*s*^ further as *I*^*s*^ increases, but we do not consider this aspect in the present work. Note that the current depends only on voltage differences, not absolute voltages, because one can always change the reference level of all voltages by the same amount without changing the dynamics [22]; however, it is useful to set V=0 to be the point at which prices change sign, which is the point at which a consumer woule be paid to take the good due to glut or similar factors. In general, linear equations such as (5) and (6) will only apply over limited ranges and nonlinear generalizations will be needed to track large variations.

If waste goods exiting at $V^{w}$ were to be recycled as raw materials for another process, an electromotive force (EMF) would have to be supplied to boost them to a sufficiently high voltage (low price) to compete with fresh raw materials; otherwise they would be dumped. This EMF represents the unit cost of the goods written off by the consumer in the process of consumption, plus any subsidies provided to ensure reuse. Of course, in some cases, direct use of waste products by another production process would be possible, starting at an even lower voltage $V^{d}$, without subsidy (e.g., scrap metal).

The total power *U* expended in a resistor, or by an EMF, is the product of the current through it and the voltage difference across it. So, for example,

$$U^{s}=I^{s}(V^{s}-V^{m}),$$ (7)

$$U^{d}=I^{d}(V^{m}-V^{d}),$$ (8)

$$U^{w}=I^{d}(V^{d}-V^{w}),$$ (9)

with $I^{d}=I^{s}$ in this case. Equation (7) gives the producer’s rate of profit, which is total sales income per unit time minus the rate of expenditure on raw materials and other costs of production. Equation (8) is a measure of the rate at which value is written off during consumption, while Eq. (9) is the further loss of value to waste.

### 2.3 Production and consumption of goods

External factors affect supply and demand. For example, production may be sold only at a specified price (e.g., approximately as is done for gem-quality diamonds where suppliers form an oligopoly) and supply and demand will adjust accordingly. In this case $V^{s}$ is specified in Eqs (1) and (2) and *I*^*s*^ is determined from it, with a positive flow only if $V^{s}>V^{d}$; i.e., if the production price is less than the most the consumer will pay. In this case $V^{s}$ is a voltage source whose time dependence, if any, is specified. Because the model is linear, a negative *I*^*s*^ is possible in principle, but this could be prevented by adding a diode to prevent reverse currents into the production sector (i.e., returns of goods to the producer). In the present work, we assume that $V^{s}>V^{d}$ to avoid such effects.

An alternative situation is if production is at a specified rate *I*^*s*^ (e.g., as for perishable fruit), a current source, and the prices $-V^{s}$ and $-V^{m}$ must adjust to satisfy Eq. (1), rising if *I*^*s*^ falls and vice versa. In this case, the voltage sources in the top left frame of Fig 1 should be replaced by current sources, subject to conservation of current.

On the demand side, there may likewise be a specified voltage sink $V^{d}$, which need not be constant in time (e.g., the price commanded by winter clothing goes down in summer), or there may be a specified rate of consumption (e.g., of basic foodstuffs) corresponding to a current sink *I*^*d*^. In both cases, Eqs (1) and (2) will still be satisfied.

### 2.4 Impedance model

In the above purely resistive model, currents and voltages can depend on time, but all responses are instantaneous, which is unrealistic. Hence, we extend the model to include dynamical effects that can affect flows of goods. The bottom left frame of Fig 1 shows a time-dependent situation in which changes in price are opposed by producer and consumer inertia, represented by the inductances *L*^*s*^ and *L*^*d*^, which produce inductive voltages of the form *LdI*/*dt* that oppose changes in currents *I*. This effect models inertia in flows of goods because of contractual obligations, items being in transit, and goods being in the process of being produced or consumed, for example, all of which oppose sudden changes in flow rates. Stockpiles of product can be modeled by including capacitances such as the ones in the *r* branch in the middle bottom frame of Fig 1. The producer also accumulates an amount of inventory *Q*^*s*^ in the *r* branch, given by

$$Q^{s}=C^{r}(V^{s}-V^{r})=C^{s}I^{r}/G^{r},$$ (10)

in the steady state. In the architecture seen in the top right frame of Fig 1 any electric charge (goods) that flows onto the capacitor forces an equal charge off the opposite plate, so the overall flow to the consumer is always *I*^*s*^. However, if $V^{s}$ is replaced by a source *I*^*s*^ in the modified architecture in the bottom middle frame of Fig 1, a reserve price $-V^{r}$ is set by the producer, below which Eq. (10) implies that they stockpile some of their production by charging the capacitor *C*^*r*^ (the bigger *C*^*r*^ the more ability there is to stockpile), with only the remainder going to market. If prices rise above the reserve, an outward flow of stockpiled goods adds to the instantaneous production rate to supply the market. These effects tend to smooth market fluctuations under time-varying conditions, while taking advantage of any transiently higher market prices (thereby potentially modeling some aspects of price anticipation), with no effect on prices in the steady state, while the resistance *R*^*r*^ represents costs incurred to move goods to and from storage. Stockpiling by consumers could also be included in a similar way.

In the time-dependent case, it is convenient to Fourier transform all the quantities and work with angular-frequency ω in place of time *t*, with

$$g(\omega )=\int g(t)e^{-j\omega t}dt,$$ (11)

being the Fourier transform of a function *g*(*t*), and the inverse transform given by

$$g(t)=\int g(\omega )e^{j\omega t}\frac{d\omega }{2\pi }.$$ (12)

Henceforth, we assume that all quantities are written in Fourier space unless the argument *t* is shown. In Eqs (11) and (12), *j* is the square root of –1 with positive imaginary part, written as *j* in electrical circuit theory to avoid confusion with currents that are often written with lower case *i*. (Many branches of physics write temporal variations in the form $e^{-j\omega t}$, especially for wave propagation problems in plasmas and optics, but we follow the electrical convention here.) In this representation a resistance has an impedance *Z* = *R* and a capacitance has impedance Z=−j/(ωC) which has a phase lag relative to *R* because capacitors take time to charge. The inverse dependence on ω reflects charging and the fact that capacitors cannot pass direct currents (ω=0). An inductor has an impedance jωL which reflects that its effect increases with frequency to oppose rapid changes, as discussed above, and thus leads the resistive effects in phase. Impedances are combined in series and parallel in the same way as resistances [21].

Overall, the generalization of resistance *R* to impedance *Z* allows for the fact that the response of current to voltage changes is not instantaneous, but can lead or lag the applied voltage in phase in the Fourier domain. For example, the opposition of inductive effects causes currents to lag, whereas currents must flow for some time before capacitive voltages build up, so they lead the voltage, which explains how they can incorporate aspects of price anticipation.

It is common to write the overall impedance of a circuit block as [21]

Z=R+jX, (13)

where *X* is termed the reactance, which is inductive if its phase is positive and capacitive if it is negative. In many calculations it is useful to write the equations in terms of the admittance *Y* = 1/*Z*, which generalizes the conductance *G* to include phase in equations (5) and (6), for example, and is shown in the bottom right frame of Fig 1. In this context, admittance is closely related to a generalized price elasticity that incorporates a relative temporal lag or lead and can also be used to represent internal structures of the production and consumption sectors other than those shown in the bottom left frame of Fig 1. For illustrative purposes below, we note that the impedance of a single block in this frame has the form

Z=R+jωL, (14)

which approaches *R* in the static case as ω→0.

Before leaving this section, we briefly summarize the quantities and units used in the model and their electrical analogs in Table 1. Note that we follow the standard convention that quantities are written in italic font, while units are in non-italic font—this avoids confusion between capacitance (*C*) and coulombs (C), for example.

**Table 1:** Dynamic quantities and parameters shown above and below the line, respectively, with their symbols in the second column, and units in the third, where $ denotes money, T denotes a unit of time (e.g., years), and Q is the unit of goods. Note that −V is unit price. Most terminology for model economic quantities is taken directly from the electrical analogs in columns four to six, especially where no specific term for the relevant quantity exists in economics. An alternative would be to use terms such as economic inertia, storage capacity, etc., for inductance, capacitance, etc., in column 4.

Quantity	Symbol	Unit	Analog	Symbol	Unit
time	*t*	T	time	*t*	second (s)
value	*E*	$	energy	*E*	joule (*J*)
goods	*Q*	Q	charge	*Q*	coulomb (C)
unit price	*P*	$/Q, V	voltage	V	volt (V)
goods flow	*I*	A, Q/T	current	*I*	ampere (A)
friction	*R*	Ω, V/A	resistance	*R*	ohm (Ω)
impedance	*Z*	Ω, V/A	impedance	*Z*	ohm (Ω)
conductance	*G*	S, A/V	conductance	*G*	siemens (S)
admittance	*Y*	S, A/V	admittance	*Y*	siemens (S)
storage	*C*	F, C/V	capacitance	*C*	farad (F)
inductance	*L*	H, VT/A	inductance	*L*	henry (H)
money flow	*U*	$/T	power	*P*	watt (W)

## 3 Multi-location impedance network model with trade

We now extend the model of Section 2 to trade between locations, linked by trade nodes to form a discrete network. We use this approach to analyze trade flows in response to price differences between market nodes, the emergence of supply and demand curves, elasticities of prices, and price changes. This provides the basis for the numerical examples in Section 4. In the present work, we omit the *r* sector seen in the bottom middle frame of Fig 1 and use the architecture shown in the bottom left frame at each location.

### 3.1 Trade network

**Fig 2:**
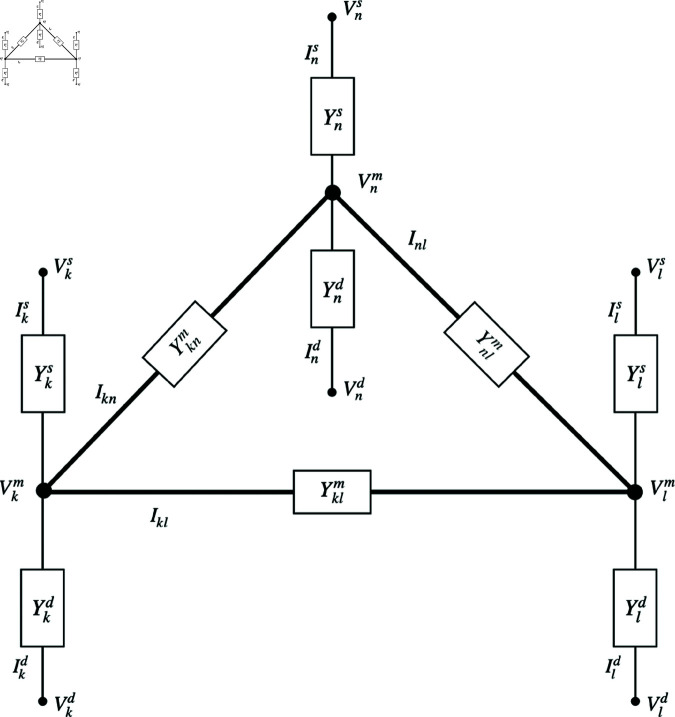
Multi-location electric circuit model of production, consumption, and trade between locations labeled k, l, and n. Nodal structure is as in the bottom left frame of Fig 1 and market nodes *k* and *l*, for example, are linked by an admittance $Y_{kl}^{m}$ through which the trade current $I_{kl}^{m}$ passes. The market price at location *k* is $-V_{k}^{m}$.

Geographical locations can be anything from different localities within a country to different countries, depending on the desired spatial resolution. We label geographical locations with the subscript k=1,…,N The internal structure at each location is as in the top right frame of Fig 1, but the locations are now linked by admittances $Y_{kl}^{m}=Y_{lk}^{m}$ that connect market nodes at locations *k* and *l*, with the market node at location *k* having voltage $V_{k}^{m}$, as shown in Fig 2. Use of symmetric link impedances implies that all links are bidirectional—trade can equally well go in either direction. In the real world this is not always the case; e.g., a coal export terminal does not usually have the facilities to import coal. However, we restrict attention here to cases in which goods can flow both ways (e.g., via container terminals or in the case of intangible goods such as computer software). Note that $Y_{kl}=1/Z_{kl}$ embodies resistance that causes voltage drops (price increases) due to shipping and other costs of trade. Border costs such as taxes and tariffs are easy to implement in this model by increasing *R*_*kl*_ accordingly. However, trade quotas are a nonlinear effect that is not covered by the present linear analysis but could be included by means of nonlinear circuit elements. Similarly, asymmetric trade links could be treated by replacing each existing link by two links, each including a diode to allow flow only in a single direction, and each with its own impedance.

### 3.2 Trade dynamics

Once locations are interconnected by the trade network shown in Fig 2, we can determine the voltages (negative prices) and currents (flows of goods) at all points. This will enable us to interrelate supply, demand, and trade, calculate their mutual sensitivities and elasticities, and analyze their fluctuations. Being linear, the system has a unique solution for any specified boundary conditions—i.e., the external voltages at the boundaries of the system (or, equivalently, the external currents, or a self-consistent combination of currents and voltages).

Standard methods of solution exist for solving for currents and voltages in cases such as our 3*N*-node network. The *N* equations are obtained from the imposition of conservation of charge (goods) at market nodes, which implies current balance at each node, with no net inward or outward current. A total of 2*N* boundary conditions must be imposed, either in the form of specified voltage sources $V_{k}^{s}$ and $V_{k}^{d}$, corresponding current sources, or a mixture of both. In the remainder of the present work we consider boundary conditions on the prices (voltages) to keep the scope manageable.

**Fig 3:**
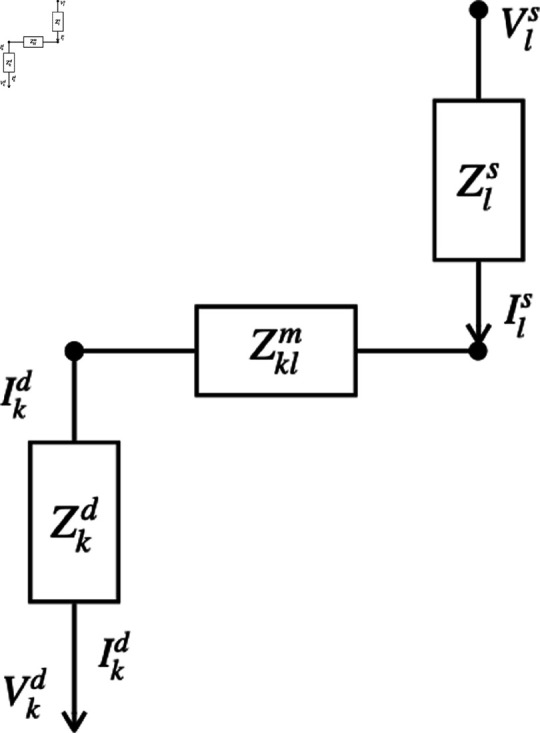
Flow to a location k from a node l via a trade network with impedance ${𝒵}_{kl}^{m}$.

We employ the Simscape physical modeling features of the Simulink graphical circuit analysis software on Matlab to solve our network problem [20]. Once the initial and boundary conditions are specified, this package integrates the time-domain circuit equations forward in time while imposing a specified error tolerance. At nonzero frequency, the total impedance $Z_{kl}$ measured between inflow of goods to the network (production) at location *l* and outflow (consumption) at node *k* can be determined by considering the elementary problem shown in Fig 3 because our system is linear, with the general solution for the flow of goods being given by the total of elementary solutions summed over all *k* and *l*. In this case

$$Z_{kl}=Z_{k}^{d}+Z_{kl}^{m}+Z_{l}^{s},$$ (15)

$$=\frac{V_{l}^{s}-V_{k}^{d}}{I_{k}^{d}}.$$ (16)

Equation (16) actually defines $Z_{kl}$, which can be determined from the numerical calculations. Then the known values of $Z_{l}^{s}$ and $Z_{k}^{d}$ can be used to calculate $Z_{kl}^{m}$, which is the impedance between the trade network nodes at *k* and *l*. In these equations, calligraphic font indicates quantities that include the effects of all direct and indirect paths that goods might take, while italic font indicates direct connections only. Equivalently, one may evaluate

$${𝒵}_{kl}^{m}=\frac{V_{l}^{m}-V_{k}^{m}}{I_{kl}^{m}},$$ (17)

where $I_{kl}^{m}$ is the current to *k* from *l* within the trade network. Steady state quantities are calculated in a similar way, with the voltages at external terminals fixed at specified, nonzero values.

Note that the trade level of the network is potentially densely connected over both long and short distances, whereas each production and consumption node has only a single connection to the trade network via its corresponding market node. Hence,

$$V_{l}^{s}=V_{l}^{m}+Z_{l}^{s}I_{l}^{s},$$ (18)

$$V_{k}^{d}=V_{k}^{m}-Z_{k}^{d}I_{k}^{d}.$$ (19)

### 3.3 Flows of goods

Once the values of ${𝒵}_{kl}$ between producers and consumers have been calculated, we can determine the production, consumption, and trade currents that represent flows of goods in the network.

The individual current *i*_*kl*_ to consumer *k* from producer *l* is given by

$$i_{kl}={𝒴}_{kl}(V_{l}^{s}-V_{k}^{d}),$$ (20)

where $Y_{kl}=1/Z_{kl}$; Eq. (20) means that the demand for goods is proportional to the difference between the price implied by the consumer’s marginal utility and the marginal cost of production; hence, supply and demand and the resulting demand curve are linear in both producer and consumer prices, which is locally reasonable but would need to be modified for large price variations. Because the system is linear, the total consumption and production currents at locations *k* and *l*, respectively, are thus

$$I_{l}^{s}=\sum_{k}i_{kl},$$ (21)

$$I_{k}^{d}=\sum_{l}i_{kl},$$ (22)

with the total network-wide supply and demand [which are equal, if the latter includes stored goods as in the bottom left frame of Fig 1] obtained by summing over the remaining subscript in Eq. (21) or Eq. (22). This then enables market shares of production and consumption to be determined.

### 3.4 Derivation of the gravity model

In this section we derive the gravity model of the dependence of trade between pairs of countries on their geographic separation from an underlying mechanistic basis and compare the results with published data on pairwise trade flows. Here we consider two cases. The first, simpler case considers how goods flow through a trade network if consumption is negligible along the way; the second includes the effect of intermediate consumption or wastage and reduces to the first in the appropriate limit. We note that existing fits of the gravity-model to trade data have implicitly averaged out differences between the properties of individual pairwise trade links. Hence, our derivation assumes average properties of all links in order to make the analysis tractable. Nonuniformities in properties could be included in numerical analysis via Simulink if their parameters could be estimated.

#### 3.4.1 Trade without consumption or wastage

**Fig 4:**
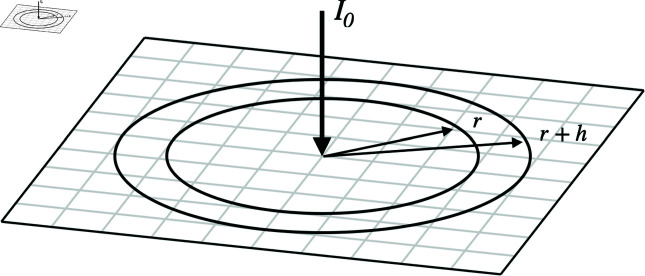
Schematic of inflow *I*_0_ of goods at *r* = 0 with consumption at various r as the product flows outwards through the market links. Nodes are shown on a square grid, separated by a distance *h*.

The total flow of goods $I_{kl}^{m}$ to location *k* of the trade network from location *l*, as shown in Fig 4, is given in terms of the trade-network impedance $Z_{kl}^{m}$ by rearranging Eq. (17). If we neglect Earth’s curvature, we can view the trade network as being located on a two-dimensional (2D) plane, to represent geography. We also approximate the network as being so finely structured as to be approximately continuous on the scale of Earth as a whole. Then a current injected at the origin gives rise to a current density *J*^*m*^ at a distance *r* of the form J∝1/r because conservation of current implies that $2\pi rJ^{m}(r)$ is constant. In this approximation

$${𝒴}_{kl}^{m}\propto 1/r,$$ (23)

where *r* is the geographical distance between locations *k* and *l*; in the resistive case, for example, this corresponds to transport costs being proportional to distance, which is approximately true for total trade between countries [6,7].

Now if a group of *N*^*s*^ geographically neighboring producer nodes are grouped together into a country (this could equally well be a region within a country), with a common $V^{s}$ for simplicity, and a group of *N*^*d*^ geographically neighboring consumer nodes are also grouped together as a country with a common $V^{d}$, application of Eqs (20)–(22) implies that the production and consumption admittances are proportional to *N*^*s*^ and *N*^*d*^ respectively and total current between these two groups is of the form

$$I_{\text{tot}}=\sum_{k}\sum_{l}i_{kl}.$$ (24)

If we assume without loss of generality that we have divided the system into nodes of equal size, *i*_0_, then

$$I_{\text{tot}}\approx i_{0}N^{s}N^{d}{𝒴}_{kl}(V_{l}^{s}-V_{k}^{d}),$$ (25)

$$\propto \frac{N^{s}N^{d}}{r},$$ (26)

where *i*_0_ is an individual current, each sum in Eq. (24) extends only over the nodes in the relevant country, and Eq. (26) should be a good approximation at distances where there are so many potential trade routes that the discreteness of the network can be ignored. Steady-state values are obtained by setting all input and output voltages and currents to constant values and thus ω=0; in this case the network is purely resistive since inductances have no effect and capacitances correspond to open-circuits that pass no current.

Equation (26) is a special case of the phenomenological gravity model of trade between countries, where *N*^*s*^ and *N*^*d*^ are proportional to the sizes of their respective economies [5,6]. Note that the exponents of the quantities in this equation are all of unit magnitude, whereas fits to data deviate somewhat from this simple approximation, albeit with significant uncertainties because of large scatter in the data due to factors other than distance, such as cultural, historical, regulatory, geographic, and political differences between different pairings of trade partners [5,6]; typical values range from 1.0 to 1.2 for the dependence on the sizes of the economies and from −0.5 to −1.4 for the dependence on distance. The theory is thus consistent with the observations, especially given the uncertainty in the observed exponents.

### 3.5 Trade with consumption or wastage

We now generalize the gravity model treated in Section 3.4 to allow for consumption or wastage at various intermediate nodes. In this case we assume the individual regions are spaced a distance *h* apart with purely resistive links *R*^*d*^ to a constant consumer voltage at $V^{d}$, resistances *R*^*m*^ between adjacent market nodes, and a current input *I* at *r* = 0, as shown in Fig 4. Since we are interested in the case when *h* is very small relative to the size of the system, we ignore current flows in the azimuthal direction because symmetry implies that these approach zero as h/r→0. We thus consider only radial currents in the plane.

If we now consider a particular location, it will have a consumption current

$$i^{d}(r)=\frac{V^{m}(r)-V^{d}}{R^{d}},$$ (27)

which gives a total consumption current in an annulus of width *h* at radius *r* of

$$I^{d}(r)=\frac{2\pi r}{h}\frac{V^{m}(r)-V^{d}}{R^{d}},$$ (28)

where the factor 2πr/h is the number of consumption nodes within the annulus for small *h*, with each taking up an area *h*^2^.

Conservation of current across the annulus implies that the total current at the market (trade) level satisfies $I^{m}(r+h)=I^{m}(r)-I^{d}(r)$, so

$$\frac{\partial I^{m}(r)}{\partial r}\approx -\frac{I^{d}(r)}{h},$$ (29)

$$=-\frac{2\pi r}{h^{2}}\frac{V^{m}(r)-V^{d}}{R^{d}},$$ (30)

where we have written $I^{m}(r+h)-I^{m}(r)\approx h\partial I^{m}(r)/\partial r$ in (29) and have used (28).

Now we also note that the current through one market link is

$$i^{m}(r)=\frac{V^{m}(r)-V^{m}(r+h)}{R^{m}},$$ (31)

so, with similar approximations to those employed in obtaining Eq. (30),

$$I^{m}(r)=-\frac{2\pi r}{R^{m}}\frac{\partial V^{m}(r)}{\partial r}.$$ (32)

where we have noted that the number of market links crossing each boundary of the annulus approaches 2πr/h for small *h*. If we then substitute Eq. (32) in to Eq. (29), we obtain

$$\frac{1}{r}\frac{\partial }{\partial r}\left(r\frac{\partial V^{m}(r)}{\partial r}\right)=\frac{R^{m}}{R^{d}h^{2}}[V^{m}(r)-V^{d}],$$ (33)

or, equivalently,

$$\nabla^{2}U(r)=\lambda^{2}U(r),$$ (34)

$$U(r)=V^{m}(r)-V^{d},$$ (35)

$$\lambda^{2}=R^{m}/(R^{d}h^{2}),$$ (36)

where $\nabla^{2}$ is the planar Laplacian.

For a source current *I*_0_ at *r* = 0, Eq. (34) has the solution [23]

$$U(r)=\frac{I_{0}R^{m}}{2\pi }K_{0}(\lambda r),$$ (37)

where *K*_0_ is a Macdonald function, which falls off approximately in proportion to $r^{-1/2}e^{-\lambda r}$ at large *r* [23]. This corresponds to the market voltage approaching the consumer voltage at large *r* as the flows become small.

Equation (32) then implies

$$I^{m}(r)=I_{0}\lambda rK_{1}(\lambda r),$$ (38)

where *K*_1_ is also a Macdonald function [23], which also decreases roughly exponentially at large *r*. Hence, from Eqs (31) and (32),

$$i^{m}(r)\approx \frac{\lambda hI_{0}}{2\pi }K_{1}(\lambda r),$$ (39)

At large *r*, the roughly exponential fall-off reflects the consumption of a fraction of the flow in each successively larger annulus as *r* increases.

Similarly, from Eqs (27) and (37) we find

$$i^{d}(r)=\frac{R^{m}I_{0}}{2\pi R^{d}}K_{0}(\lambda r).$$ (40)

Hence, by considering the consumption flow out of all the nodes in an annulus of width *h*, we find

$$I^{d}(r)=\frac{rR^{m}I_{0}}{hR^{d}}K_{0}(\lambda r).$$ (41)

When consumption is small (*R*^*d*^ large), the argument of *K*_1_ is small and $K_{1}(z)\approx 1/z$, with $z=(r/h)\sqrt{R^{m}/R^{d}}$. Hence,

$$i^{m}(r)\approx \frac{I_{0}h}{2\pi r},$$ (42)

consistent with Eq. (26). The crossover from 1/*r* to exponential decrease occurs at z≈1, which corresponds to

$$r\approx 1/\lambda =h\sqrt{R^{d}/R^{m}}.$$ (43)

The above results imply that the usual power-law gravity model will still apply at moderate *r*, with a crossover to an approximately exponential decrease at large *r*; this may explain slightly higher exponents in fits to some data sets [5,6]. We explore these results numerically in Section 5.1.

An extremely important point to note regarding this section and the previous one is that, although voltages fall (prices rise) with distance, this does not open up the possibility of profiting through arbitrage. Rather, the market price at each location is struck self-consistently after allowing for the friction implicit in transport and other costs involved in moving goods from producers to market at a different location. This also applies in the case of multiple producers and trade routes; if one producer has low production and transport costs via a particular trade route, they will sell more goods at a given location than one with a higher cost base, but at the same market price, which is determined by all producers’ and consumers’ influences collectively. A further key advantage of the present model is that it automatically includes all possible paths between producer and consumer and the flows along them.

### 3.6 Sensitivities, elasticities, supply and demand

Having calculated the $Z_{kl}$, we can now compute sensitivities of dependent market prices and flows to independent quantities such as imposed producer and consumer prices, imposed supply and demand levels, and the properties of trade links. These are usually expressed as fractional sensitivities, or elasticities, with the elasticity of *A* with respect to changes in *B* being defined as [24]

$$E(A,B)=-\frac{\partial \ln A}{\partial \ln B},$$ (44)

$$=-\frac{B}{A}\frac{\partial A}{\partial B},$$ (45)

where the minus sign appears because a decrease in the quantity demanded (*A*) due to an increase in price (*B*) is conventionally termed a positive elasticity. A further convention is that changes with *E*_*AB*_>1 are termed elastic, whereas cases with *E*_*AB*_<1 are termed inelastic. Elastic demand relationships mean that the quantity demanded falls rapidly as price increases whereas the quantity demanded for essential goods changes only slowly with price and is thus inelastic. Note that Eqs (44) and (45) enable a broad variety of generalized elasticities to be defined, such as the elasticity of demand relative to producer price, rather than simply to the market price. In many respects, elasticities with respect to producer and consumer quantities are more fundamental than the more commonly considered elasticities with respect to market prices, which are ultimately functions of producer and consumer quantities.

The derivatives of production and consumption with respect to market prices at their locations are simply obtained from the multilocation versions of Eqs (5) and (6), with admittances also replacing conductances. This gives

$$-\frac{\partial I_{l}^{s}}{\partial V_{l}^{m}}=Y_{l}^{s},$$ (46)

$$-\frac{\partial I_{k}^{d}}{\partial V_{k}^{m}}=-Y_{k}^{d},$$ (47)

which can be substituted into Eqs (44) and (45) to obtain the corresponding elasticities.

Similarly, the derivatives of demand to changes in producer and consumer prices are

$$\frac{\partial I_{k}^{d}}{\partial V_{l}^{s}}=Y_{kl}=-\frac{\partial I_{k}^{d}}{\partial V_{l}^{d}},$$ (48)

from Eq. (17). Equation (48) implies that an increase in consumption will tend to occur if $V_{l}^{s}$ increases (i.e., if the marginal cost of production decreases), thereby expressing the relationship between supply and demand; likewise, a decrease in supply will follow from a decrease in the consumer’s marginal utility. Note that if overall impedance ${𝒵}_{kl}$ between producer and consumer is high—e.g., because trade costs (i.e., resistances $R_{kl}^{m}$) are high—the admittance in Eq. (17) will be small and the producer *l* will be largely unaffected by consumer *k* unless the voltage difference in Eq. (17) is very large (i.e., unless a sufficiently high price is paid by consumer *k* to attract a significant flow of goods from *l*).

We can calculate the flows through the individual direct trade links between locations, which are related to transport capacity. Specifically, having calculated the $V_{k}^{m}$, we find

$$I_{kl}^{m}=Y_{kl}^{m}(V_{l}^{m}-V_{k}^{m}).$$ (49)

The above results show that flow in a link does not directly measure its capacity (i.e., its $Y_{kl}^{m}$), but also depends on the price difference across the link in question and thus also involves competing links because they also affect this difference. This is consistent with the more recent economics literature that discusses, not just bilateral barriers to trade, but also the idea of “multilateral resistance” terms [7,8]. Bilateral trade thus depends not just on bilateral trade barriers and friction, but also the trade barriers and friction that trade partners have relative to all other countries. Some recent trade network analyses have focused on node degrees (the number of links) and edge strengths, the latter corresponding to our link admittances, that measure network connectivity [25–27]; our approach naturally accommodates such applications.

Equation (49) also implies that trade flows whenever there is a price difference to drive it. More generally, there must usually also be a minimum return on investment, which would impose a nonlinear threshold dependency that could be included via nonlinear circuit elements. Likewise, a maximum capacity exists on each link that limits $I_{kl}^{m}$ no matter how high the price difference across the link; we also leave this nonlinear saturation for future work.

### 3.7 Profit, expenditure, and trade friction

The power dissipation in a resistive element of the circuit is [21,22]

$$U=R\langle I^{2}\rangle ,$$ (50)

where the angle brackets denote a time average, which yields a factor of 1/2 at nonzero frequencies in the Fourier domain. There are also reactive power contributions that average to zero over time so we do not discuss them here.

Equation (50) represents the rate of irreversible expenditure (frictional losses) involved in the production, storage, transport, or consumption of goods, including components to account for producer and market (trading) profits. For example, in Fig 2a one may write

$$U^{r}=R^{r}\langle (I^{r}{)}^{2}\rangle ,$$ (51)

$$U^{s}=R^{s}\langle (I^{s}{)}^{2}\rangle ,$$ (52)

$$U^{d}=R^{d}\langle (I^{d}{)}^{2}\rangle .$$ (53)

Here, Eq. (51) represents the cost per unit time of moving goods to and from storage and does not depend on the direction; Eq. (52) is the cost per unit time of production, including any profit; and Eq. (53) gives the consumer expenditure. One might think that profit should be linear in *I*^*s*^, not quadratic; however, the flow *I*^*s*^ is proportional to $V^{s}\;-\;V^{m}$, as is the unit profit, so the final result is quadratic. Similar arguments apply to Eqs (51) and (53).

In the steady state, the rate of energy dissipation in an electric circuit is the minimum possible, a result that goes back to Maxwell and has since been extended from purely resistive circuits to circuits with reactive elements [28–30]. An extremely important consequence for the present model is thus that it implies that trade flows self-organize to minimize frictional losses, subject to the boundary conditions imposed on the network as a whole—i.e., the flows are the most efficient possible. This global property is emergent from the local dynamics of individual producers, consumers, and traders and is not equivalent to the longstanding observation that trade itself can improve the global allocation of resources to production. One way to view this emergence is that overall frictional losses can always be reduced by shifting trade from suboptimal routes to ones with fewer frictional losses, until such time as a minimum-friction state is reached; electrical circuit theory rigorously implies that such suboptimal flows will decay away automatically via such readjustments, resulting in this outcome as an emergent endpoint.

## 4 Three-location dynamics

In this section and the next we continuing to generalize our model by exploring its steady states and dynamics for three-node and multi-node cases, respectively, and compare them with the above analytic results. We use the Simulink software package [20] to solve the equations of our circuit model, as shown in Fig 2 but with more locations in Section 5, to illustrate its dynamics and predictions for a variety of quantities. The time-domain approach employed in this software will facilitate further extensions of the model to include nonlinear circuit elements, for example. The present work assumes voltage sources throughout and different flows of goods are achieved by changing the externally imposed supply and/or demand voltages (prices) rather than by introducing current sources to represent fixed supply or demand, although that could be done in future work.

The three-node case considered in this section provides valuable insights into the operation of the model, especially for interdisciplinary readers. Section 4.1 discusses steady state properties before considering elasticities and sensitivities. In Section 4.2 we then explore price fluctuations and their power spectra using data from high-frequency trading of stocks and commodities after explaining how our model can potentially be applied to such situations. The default parameters of the model are listed in Table 2.

**Table 2:** Nominal parameters used in the numerical simulations, except where otherwise noted in the text, where T is the chosen unit of time, which is arbitrary here. Nominal cases are purely resistive and price is the negative of voltage.

Quantity	Symbol	Value	Unit
Timestep	Δt	0.25	T
Supply Price	$-V_{j}^{s}$	50	$
Demand Price	$-V_{j}^{d}$	70	$
Supply Resistance	$R_{j}^{s}$	1	Ω
Demand Resistance	$R_{j}^{d}$	1	Ω
Market Resistance	$R_{jk}^{m}$	1,3,4	Ω

### 4.1 Steady states and elasticities

**Fig 5:**
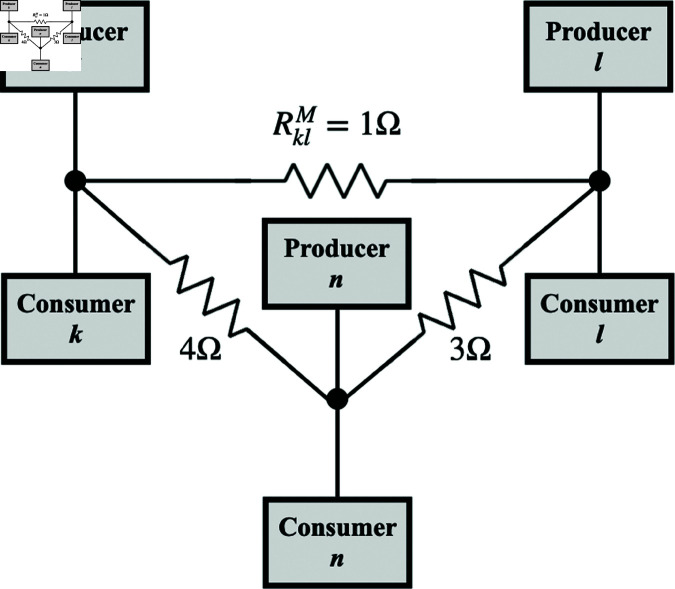
Schematic of three-location case discussed in Section 4. The resistances in the market links are as shown. Other parameters are as in Table 2.

The simplest network to have both direct and indirect market links is the three-location case shown in Fig 5. In order to verify the correct operation of the code, we have checked that: (i) The results reproduce analytic formulas for the one-location case when all other supply (producer) and demand (consumer) resistances are set to infinity to cut off the respective currents, or when all market impedances are infinite. (ii) The results reproduce analytic results for two-node cases in general and in various limits in which specific impedances are set to zero or infinity, or in which the frequency is set to zero in the Fourier domain. The set of test cases also included ones with nonzero inductive and capacitive impedances.

**Fig 6:**
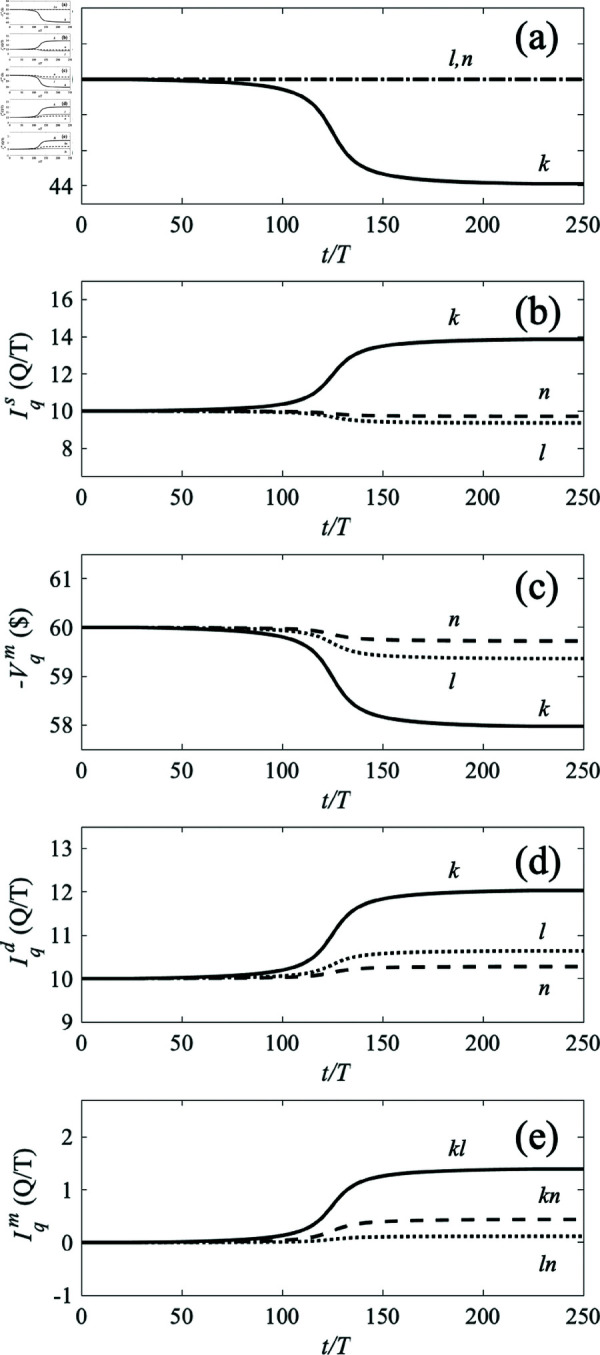
Dependence of prices (negative voltages) and currents in the three-node system shown in Fig 5 as the production (supply) price at node k is ramped down over time from $50 to $45, with consumption (demand) prices held constant at $70. As in Table 1, abbreviations for electrical units are used to represent the corresponding economic quantities. In frames (a)–(d), the solid, dotted, and dashed lines represent the currents and prices of locations q=k,l,n, respectively. (a) Supply prices $-V_{q}^{s}$. (b) Supply currents $I_{q}^{s}$. (c) Market prices $-V_{q}^{m}$. (d) Demand currents $I_{q}^{d}$. (e) Trade currents $\left|I_{kl}^{m}\right|$ (solid), $\left|I_{ln}^{m}\right|$ (dotted), and $\left|I_{kn}^{m}\right|$ (dashed).

Fig 6 shows the voltages and currents as the producer price at location *k* is ramped down after an initial constant period, while holding the other producer prices constant, as seen in Fig 6a. The initial steady-state values of all quantities are in accord with our analytic formulas. As producer *k*’s price falls (i.e., its voltage rises), its market sales increase substantially, as seen in Fig 6b. The supply from the other locations falls as they are outcompeted and substituted by producer *k*’s output; however, this effect is relatively small because of the intervening market-link resistances. Fig 6c shows that the lower producer price causes the market price to fall substantially at location *k* and less so at other locations, with a greater reduction at *l*, which is connected to *k* by a lower resistance link than the one that links *k* to *n*. In Fig 6d we see that this leads to increased consumption at all nodes, especially node *k*, in accord with the system having changed its position on the self-consistent demand curve. Trade currents, shown in Fig 6e, are initially zero but later flow outward from node *k* to the other nodes. Significantly some flow reaches node *n* via node *l*, driven by the difference in market prices between *n* and *l*, adding to the direct flow from *k* to *l*. This also implies that links can at least partially substitute for one another in the event that some are broken or have increased impedance, although any degradation of the network will tend to reduce overall trade, as in the case of widespread introduction of tariffs in the real world.

**Fig 7:**
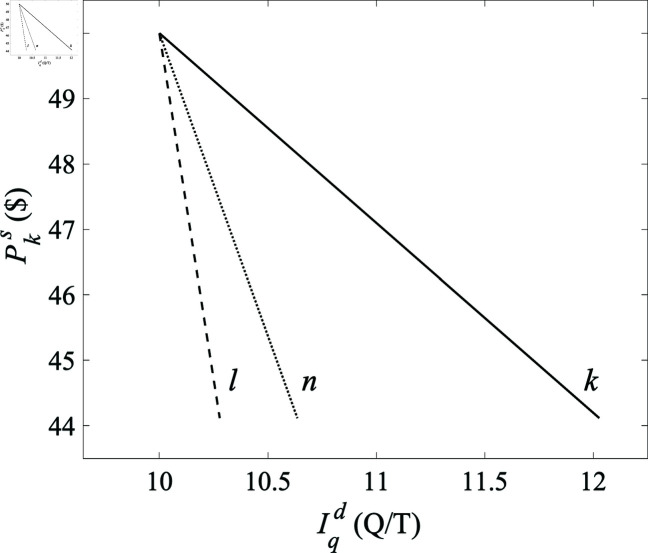
Consumer currents $I_{q}^{d}$ as the unit production price $P_{k}^{s}$ is ramped down from $50 to $45 in the three-node system of Figs 5 and 6, with consumption prices held constant at $70 and other producers’ prices fixed at $50. Currents at nodes *k*, *l*, and *n* are shown solid, dotted, and dashed, respectively, with the initial state at upper left. Following economic convention, price is on the vertical axis.

Sensitivities and elasticities can be obtained from the results in Fig 7 by comparing the changes in dependent variables to those in the control variable, which is the producer price in this case. The slopes of the lines (straight lines because of the linear approximations that are made in the present examples) in the figure yield the relevant sensitivities, whence Eq. (45) gives the elasticities $E(I_{q}^{d},V_{k}^{s})\text{for}q=k,l,n$ to be 2.90, 9.25, and 21.14, respectively; all the results agree with the formulas in Section 3.6. Elasticities between other pairs of quantities, can be obtained by systematically ramping quantities such as consumer voltages or network resistances.

### 4.2 Fluctuations

Relatively little data exists in the form of long time series of price changes of physically traded goods. However, differences between prices recorded at different times have been widely studied in high-frequency markets for stocks and commodities [31–37], and these series are long enough to study their statistics. We thus turn to these data to further explore both the properties of the model and its possible applicability to a wider class of trading problems. Before doing so, it is important to justify why it is at least plausible to try to treat this type of problem with the same model as for goods trade, despite there being no physical movement of goods: (i) as for physical goods, the greater the price difference between bids and offers, the more sales will occur; (ii) there are still multiple buyers and sellers at different locations; (iii) transfers between buyers and sellers still involve friction (resistance) in the form of taxes, commissions, and brokerage costs; and (iv) the market still acts to balance supply and demand at a market price. However, we stress that this aspect of our analysis is tentative, with the aim of exposing some of the key issues that would need to be addressed in a fuller treatment.

High-frequency price increments typically have heavy-tailed probability distribution functions (pdfs) that broaden with the size of the time difference Δt for which the price increment

$$\Delta P_{k}^{m}(\Delta t)=P_{k}^{m}(t+\Delta t)-P_{k}^{m}(t).$$ (54)

is recorded. In this section we make an initial examination of the pdfs from our model when it is driven by white noise in order to determine which of the above features are embodied in the present approximation of linear dynamics and to provide an initial illustration of spatial and inductive effects.

**Fig 8:**
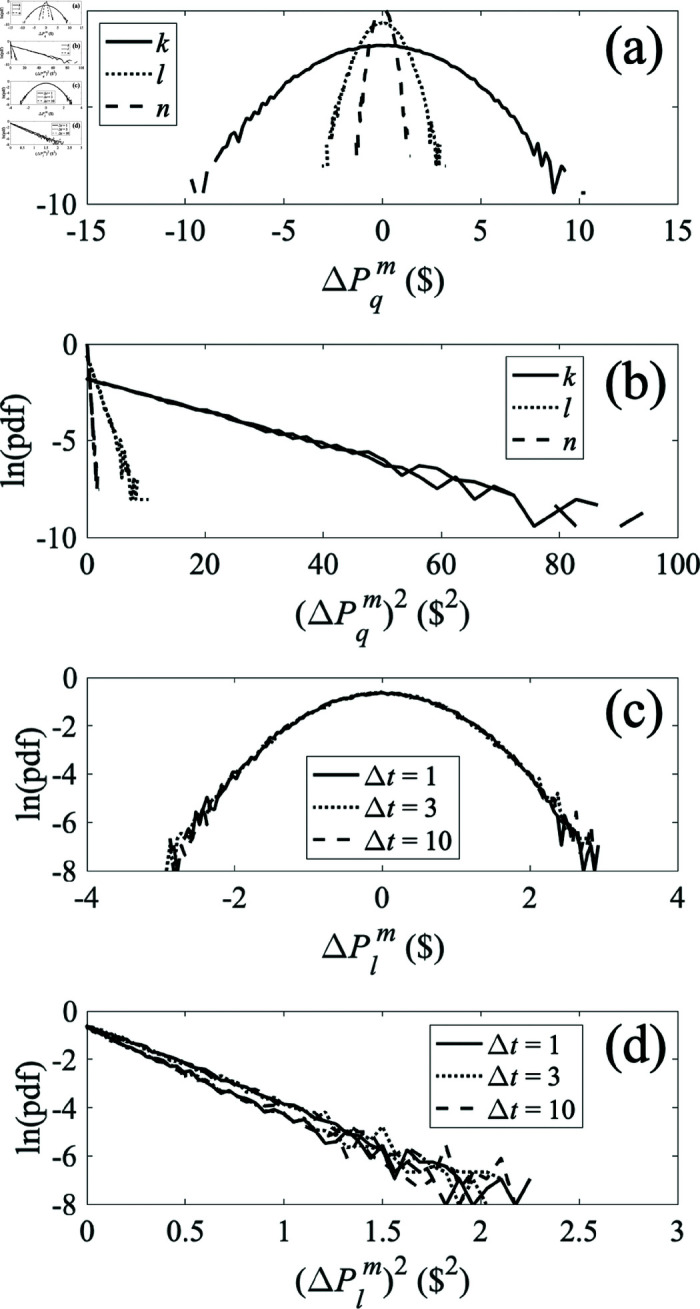
Probability density functions (pdfs) of prices for various locations q=k,l,n and time increments Δt for the three-node system shown in Fig 5. The production price $P_{k}^{s}$ at node *k* has a mean of $50 plus Gaussian white noise with a standard deviation of $5; consumption prices $P_{q}^{d}$ are held constant at $70. ( **a**) Pdf of market price increments $\Delta P_{q}^{m}(\Delta t)\text{for}\Delta t=$ T. ( **b**) Same as frame (a) but plotted vs. the squared deviation of market prices $\Delta P_{q}^{m}(\Delta t)\text{for}\Delta t=$ T. ( **c**) Pdf of market price increments $\Delta P_{l}^{m}(\Delta t)\text{for}\Delta t=1,3,10$ T. ( **d**) Same as frame (c) but plotted vs. squared deviation of market price $\Delta P_{l}^{m}(\Delta t)\text{for}\Delta t=1,3,10$ T.

Fig 8 shows pdfs of market price increments from the purely resistive system in Fig 5 when all consumers have a mean unit price $P_{q}^{d}$ of $70, all producers have a mean price $P_{q}^{s}$ of $50, and white noise with a standard deviation of $5 is added to producer *k*’s price $P_{k}^{s}$. In frame (a) we see the distributions at each node for Δt= T, where T is the unit of time, showing that the largest fluctuations occur nearest the producer that causes them, while distributions become narrower for nodes with greater intervening resistance (i.e., the effects are more localized). Hence, the market price is buffered by presence of multiple producers and consumers, a point to which we return in Section 5.2. The log-linear distributions in Fig 8a are approximately parabolic in form, which implies that they are near-Gaussian. Fig 8b shows the same results plotted against the square of the price increment, with the results for positive and negative increments plotted as separate curves. In each case the curves in Fig 8b are consistent with straight lines of the same slope for both positive and negative increments, thus indicating symmetric Gaussian distributions. Figs 8c and 8d have the same format as Fig 8a and 8b, respectively, but show price increments at node *k* at various Δt. This shows that the width of the distribution does not change in time, which is a consequence of the system being purely resistive so that currents respond instantaneously to voltage changes.

The results in Fig 8 do not exhibit the approximately power-law tails seen in high-frequency market data, nor the increase in width seen in such data as Δt increases [31–33]. As noted above, high-frequency financial market data may not be directly relevant to the trade in physical goods considered in the rest of the present work, but we consider these points further below to begin to clarify the directions in which the model would need to be generalized to cover such cases in future.

**Fig 9:**
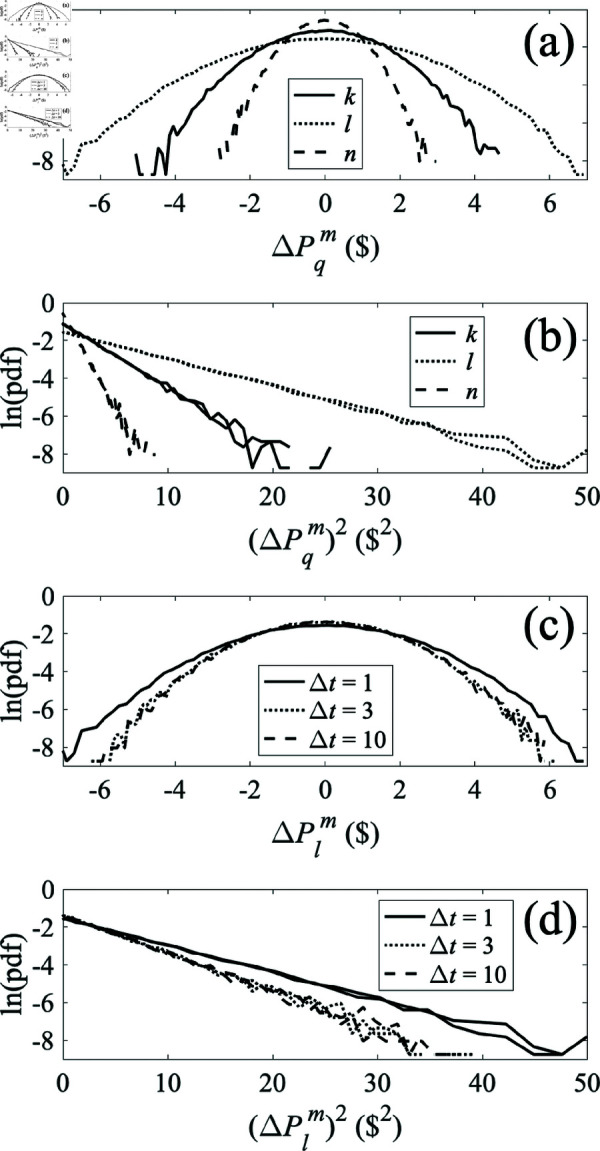
Probability density functions (pdfs) of prices for various locations q=k,l,nand time incrementsΔt for the three-node system shown in Fig 5 but with *L* = 1 H placed in series with each resistor. The production price $P_{k}^{s}\text{at node}k$ has a mean of $50 plus Gaussian white noise with a standard deviation of $5; consumption prices $P_{q}^{d}$ are held constant at $70. ( **a**) Pdf of market price increments $\Delta P_{q}^{m}(\Delta t)$ for Δt= T. ( **b**) Same as frame (a) but plotted vs. squared deviation of market prices $\Delta P_{q}^{m}(\Delta t)$ for Δt= T. ( **c**) Pdf of market price increments $\Delta P_{l}^{m}(\Delta t)$ for Δt=1,3,10 T. ( **d**) Same as frame (c) but plotted vs. squared deviation of market price $\Delta P_{l}^{m}(\Delta t)$ for Δt=1,3,10 T.

In Fig 9 we show the effect of adding an inductance *L* = 1 H in series with each resistor in Fig 5, as in the bottom left frame of Fig 1; these introduce time-dependent inertial effects that oppose rapid changes in flows of goods and our aim is to determine whether these can produce heavy tails on the pdfs and/or widths that increase in time, thereby pointing the direction for future generalizations of the model. The results in Fig 9a and 9b are very similar to those in Fig 8a and 8b, except that the width of the distribution is greater, owing to the inertia introduced via nonzero inductance *L*. Other numerical results (not shown) demonstrate that this increase is monotonic with increasing *L*. Fig 9c and 9d shows that the widths of the distributions at various locations increase with time before leveling off around Δt=3 T. As noted above, a time increase is qualitatively consistent with what is seen in high-frequency data, but there is not sufficient dynamic range available before saturation to compare with the approximate power-law dependence found in those data, and the data did not exhibit the leveling off observed here [31–33].

The results in Fig 9 indicate that system inertia is important to production of heavy tails in the pdfs. The reason that our results do not match the tails seen in high-frequency data is likely that all components in our system have standard frequency dependences of impedance (inductive contribution ∝ω and capacitive contribution ∝1/ω). Hence, the system transfer function is a rational function. However, a heavy-tailed distribution such as a Lévy stable distribution involves characteristic functions that are exponentials of fractional powers of frequency [32]. Introduction of components with fractional-power frequency dependences of impedance is thus likely to be necessary to reproduce the low-frequency behavior that would underlie scale-free memory effects in the system, but this aspect is beyond the scope of the present paper.

## 5 Multi-location dynamics

We now consider trade flows in square grids of 10×10 or 31×31 locations with each location connected only to its four nearest neighbors, e.g., as in a road network laid out in a grid. All nodes and links of each type are identical here. Many other possibilities exist for network topologies and connectivities, so the present instance should be viewed as a proof-of-principle demonstration.

In Section 5.1 we use a large network to verify our predictions for the gravity model before examining steady states and elasticities in Section 5.2 and then fluctuations in Section 5.3. The latter sections are kept relatively brief because of close similarities of many features with those of the three-node case of Section 4.

### 5.1 Gravity model

In this section we compare the gravity-model predictions of Section 3.5 with numerics by using a 31×31 grid of nodes, each separated from its nearest neighbors by a distance *h* with a single producer at the center and all parameters as in Table 1, except that the consumer resistance is reduced at *r*>12*h* in order to absorb the available supply before it reaches the system boundary—in effect, the square boundary is replaced with a near-circular absorbing boundary at *r* = 12*h*.

**Fig 10:**
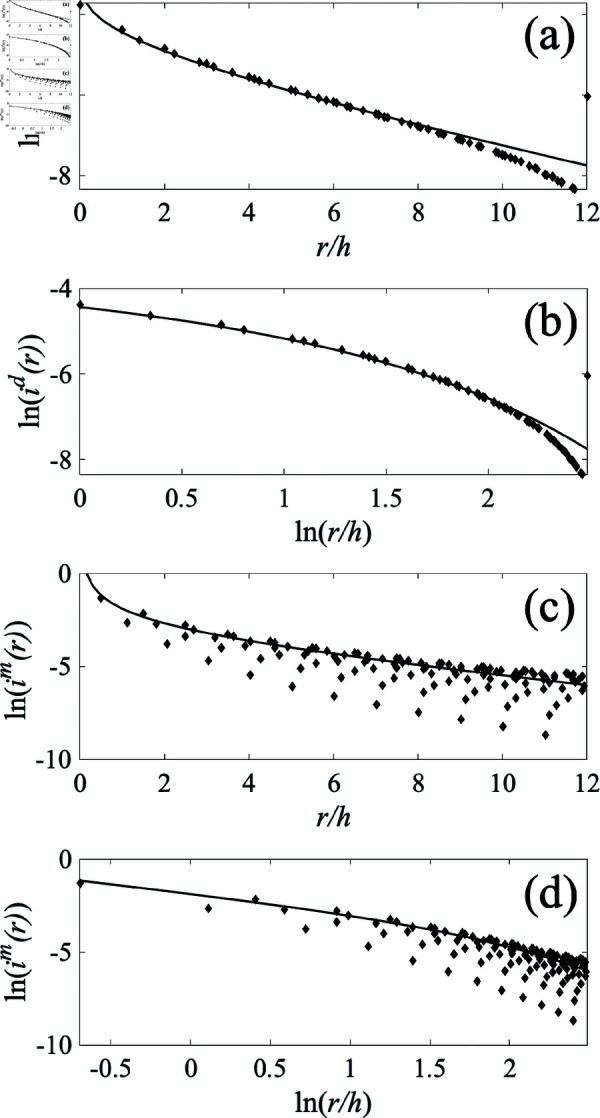
Trade flows through a 31×31 network with consumption, as shown in Fig 4. Here $R^{m}=6.5\;\Omega$, $R^{d}=150\;\Omega$, and *I*_0_ = 1 A. Plots only extend to *r* = 12*h* to avoid edge effects and numerical results are shown as diamonds. ( **a**) *i*^*d*^ vs. *r* on a log-linear scale. Equation (39) is shown solid. ( **b**) As for (a) but on a log-log scale ( **c**) *i*^*m*^ vs. *r* on a log-linear scale. Equation (40) is shown solid. ( **d**) As for (c) but on a log-log scale.

Fig 10 compares the numerical results for the consumption current *i*^*d*^(*r*) and radial market current *i*^*m*^(*r*) with the theoretical ones in Eqs (39) and (40). Logarithmic plots show the 1/*r* regime of *i*^*m*^ for r≲5h and an exponential decrease at larger *r*, with a core-periphery breakpoint consistent with the value of *r* = 5*h* predicted by Eq. (43). In these regimes the power-law exponent and slope of the exponential are consistent with the theoretical predictions out to r≈10h where edge effects start to become apparent. We have also varied this breakpoint by changing the ratio $R^{m}/R^{d}$ to verify the two regimes in more detail (not shown). Notably, the intrinsic curvature of the plots in Fig 10a and b, due to their *not* being pure power-laws, may partly explain the spread of exponents seen in the literature where the data were fitted to power laws without allowance for an exponential tail of *K*_*m*_(*z*) at large *z* nor its logarithmic variation at small *z* [5,6]. As mentioned above, observed trade volumes also depend on differences in trade friction in individual pairwise trade relationships, which contribute to wide scatter in the data.

### 5.2 Steady states and elasticities

**Fig 11:**
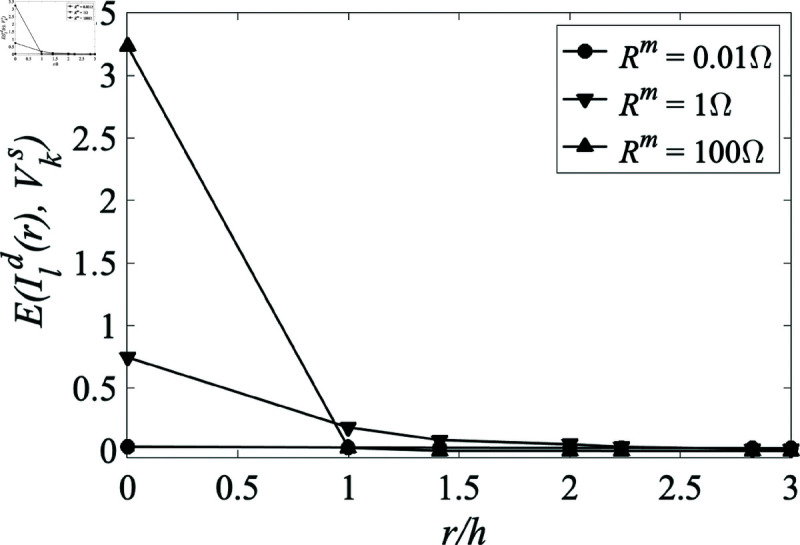
Price elasticity at consumers as a function of distance from the central producer in an 11×11 network connected with locations connected via their nearest neighbor with white noise in the centrally located producer price, with a standard deviation of $5. All locations have production prices held constant at $50 and consumption prices held constant at $70. Note that use of a square grid restricts *r* to corresponding discrete values.

In accordance with Eq. (45), the price elasticity of demand is

$$E(I_{l}^{d},V_{k}^{s})=\frac{P_{k}^{s}}{I_{l}^{d}}\frac{\partial I_{l}^{d}}{\partial P_{k}^{s}}=-\frac{V_{k}^{s}}{I_{l}^{c}}\frac{\partial I_{l}^{d}}{\partial V_{k}^{s}}$$ (55)

which is equivalent to the inverse of the slope of the classical demand curve in economics; i.e., log price vs. log quantity. Using this formula, price elasticity as a function of distance from a single producer is shown to decrease with increasing *r*, as seen in Fig 11. As the market-link resistance increases, representing increasing transport costs per unit distance, the price elasticity close to the producer rises, but falls off more rapidly with distance. In all cases, distant consumers are little affected by changes in production prices at a single producer among many, as is the case in the real world.

### 5.3 Fluctuations

**Fig 12:**
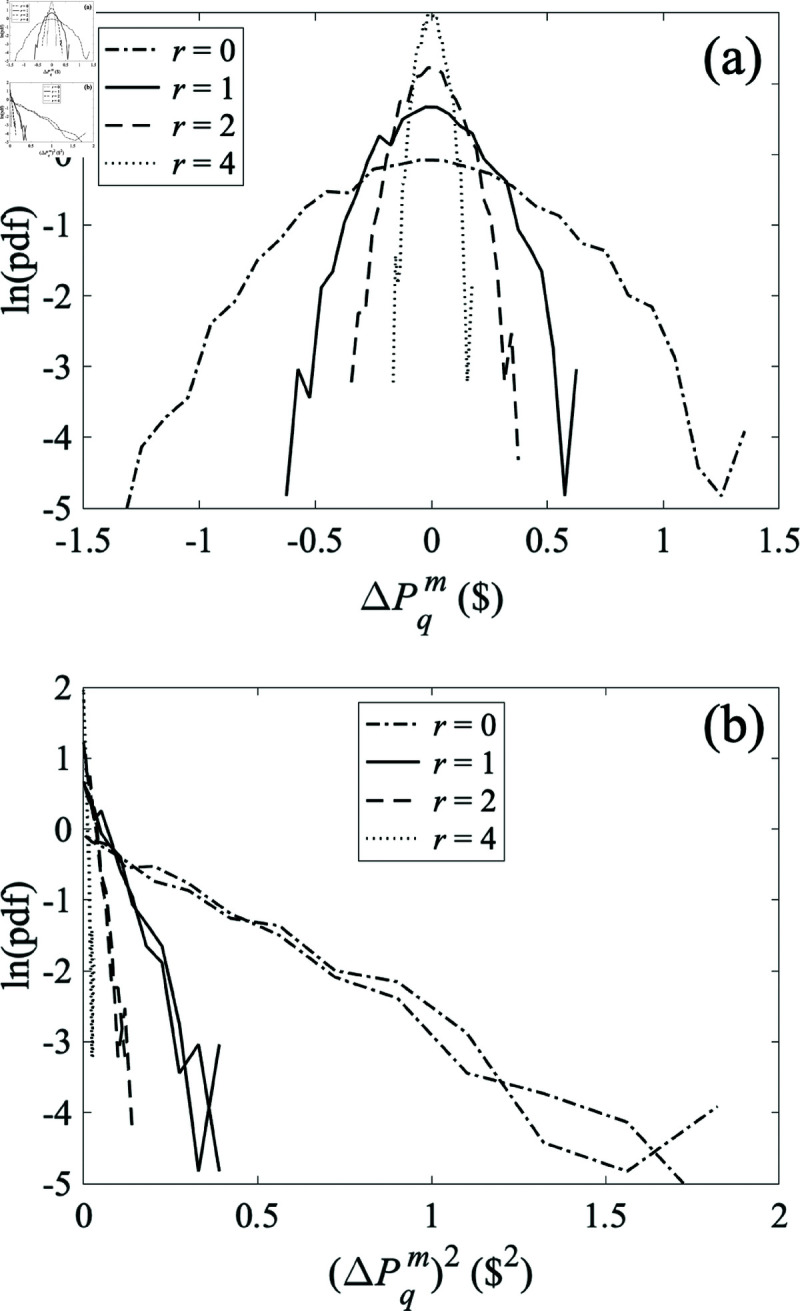
Probability density functions (pdfs) of market price increments $\Delta P_{q}^{m}(\Delta t=1)$ for locations *q* at various distances from the central producer in an 11×11 network connected with locations connected via their nearest neighbor. The production price at the central producer has a mean of $50 plus Gaussian white noise with a standard deviation of $5, while all other producers have a constant production price of $50; consumption prices are held constant at $70. Producer resistances *R*^*s*^, market-link resistances *R*^*m*^, and consumer resistances $R^{d}\text{are set to}1\Omega$, 0.1 Ω, and 5 Ω, respectively. ( **a**) Pdf of market price increments $\Delta P_{q}^{m}$ for distances r=0,1,2,4. ( **b**) Same as frame (a) but plotted vs. the squared deviation of market price $\Delta P_{q}^{m}(\Delta r)$ for Δr=0,1,2,4h.

We now consider the effect of fluctuations in the producer price on demand as a function of distance. Fig 12a shows the pdfs of demand at various distances from a producer whose production price fluctuates around $40 with a white noise offset with standard deviation of $5. We see that the pdf rapidly narrows with distance. Fig 12b plots the pdfs vs. the square of the demand, with the straight-line plots demonstrating that the distributions are Gaussian, as in the three-node case. In the units shown, the magnitudes of the slopes in Fig 12b are 0.273, 1.16, 3.81, and 16.6, respectively, which are approximately in proportion to the number M(r)of consumers within the indicated distancer from the central producer on the discrete grid. Hence, the standard deviation scales very nearly as *[M*(*r*)*]*^−1/2^.

## 6 Summary and conclusions

We have developed an initial electric circuit model of spatially distributed and time-varying supply (production), demand (consumption), and trade, focusing principally on laying the foundations of the model and verifying that it has the correct qualitative behaviors in its linear limit. This implements the first stages of the stepwise model development and verification strategy outlined in Section 2.1.

The central ansatz of the model is that the flow of goods along each link between producer and consumer, or between market nodes, increases when the corresponding price difference increases. A first tranche of results have been derived and explored from this starting point, and compared with qualitative and quantitative trade results to show that the results are consistent with data. Notably, these include derivation and generalization of the widely used gravity model of international trade and verification of the results against published data. The foundation has thus been laid for further generalization and application. The main results are as follows:

(i) We have made the analogy that trade in a single homogeneous good involves the flow of current (the amount of that good that flows per unit time), driven by differences in voltage (negative unit prices). Goods thus flow from high voltage (low price corresponding to unit production cost) at producers to low voltage (high price corresponding to unit utility) at consumers. Trade links between market nodes at different spatial locations then form a trade network. All links have resistances that cause price increases as they are traversed in either direction (trade friction, which can be increased at international boundaries to model tariffs, for example) and can also accommodate inductive and capacitive elements to reflect effects such as persistence of flows and stockpiling, respectively. In the present work the system is approximated as being linear, but we have outlined how this assumption can be relaxed.
(ii) The electrical analogy enables standard engineering software to be used to solve for currents and voltages, given suitable boundary conditions; we have used the Simscape package within Simulink [20]. Boundary conditions can be specified in terms of prices (negative voltages) that correspond to marginal cost of production and marginal consumer utility. Such software can also incorporate alternative boundary conditions such as specified supply and/or demand, and can treat nonlinear effects such as asymmetric trade links and limits on link capacity.
(iii) The model correctly implies that market prices are determined as dependent variables that are set by the dynamically determined balance of supply and demand, as in the real world, subject to system characteristics and boundary conditions. Thus, a demand curve emerges rather than needing to be separately specified.
(iv) The abilities of producers, consumers, or trade links to substitute for one another emerge directly from the model, yielding new mutually consistent flows and prices when network elements or boundary conditions are changed for whatever reason, including changes in consumer preferences, production costs, and trade barriers.
(v) Through appropriate interpretation of electrical quantities, we derived expressions for price sensitivities and elasticities of supply and demand, including novel types of elasticity. Sensitivities to the properties of trade links can also be derived within this framework, enabling the effects of bilateral trade policy settings to be evaluated.
(vi) Expressions were obtained for profits and for losses due to trade friction. Notably, these are quadratic in the voltage (negative-price) differences.
(vii) An important consequence of the minimum-dissipation theorem from electrical circuit theory is that trade flows arrange themselves to minimize frictional losses, subject to the imposed boundary conditions on the network as a whole. In other words, the trade flows self-organize to satisfy the boundary conditions on production and consumption as efficiently as possible—a result that emerges from the local dynamics of individual producers, consumers, and traders.
(viii) We analytically predicted prices and trade flows vs. distance between producer and consumer in a simple approximation that includes average trade friction. This yielded a particular version of the gravity model of trade as a direct consequence of our central ansatz. Specifically, it was found that trade flows decrease approximately inversely with distance, as widely discussed empirically, before transitioning to an exponential fall-off in the presence of consumption or wastage, which had not been previously discussed. This gives rise to a natural core-periphery structure, as seen in multinational trade.
(ix) A three-location system was used as a foil to illustrate and verify the results in the simplest nontrivial case. This enabled the results and operation of the numerical code to be checked and debugged in cases that could also be easily visualized. Among the results illustrated were the roles of alternate trade routes in buffering prices and trade volumes when one route is restricted or blocked—i.e., link substitution.
(x) It was shown how elasticities can derived numerically by slowly ramping independent variables and observing the resulting changes in dependent ones.
(xi) In an initial tentative exploration of time-dependent cases, fluctuating supply was shown to produce Gaussian distributions of price increments, whose width increased with time interval when inductive effects were included, but quickly leveled off. These results show the need to incorporate longer-memory circuit elements if observed heavy-tailed distributions that broaden over a wide range of time intervals are to be reproduced. Note that the comparison with probability distributions was with data from high-frequency trading of stocks and commodities in the absence of adequate analogous data for international physical goods trade. This requires further investigation, but the commonality of features implies that this is a promising route for further research.
(xii) Numerical analysis of larger systems of up to 961 trading locations, together with data that underlie empirical gravity models, enabled numerical confirmation of our theoretical predictions regarding the gravity model, including both the inner power-law dependence and the long-range approximately exponential fall-off in trade volumes. Comparison with published data on gravity-model exponents show good agreement, thereby further supporting the validity of the present model. Differences in parameters of individual trade links presumably account for much of the scatter in the data, and could potentially be constrained by requiring consistency with the observed trade volumes, with electric circuit theory able to account for multiple potential paths from producer to consumer.
(xiii) Elasticities of demand relative to producer price in large systems are high close to a given producer in large systems with high transport costs (large *R*^*m*^), but fall off very rapidly with distance. Elasticities are low if transport costs are low, reflecting the ability of consumers to source goods from other producers in an emergent core-periphery structure.
(xiv) Fluctuations in large systems are qualitatively similar to those seen in the three-node case, displaying Gaussian pdfs. The variance of the pdf a distance *r* from a given producer was found to fall off approximately inversely with the number of consumers within a circle of radius *r*.

As mentioned at various points in the text, many generalizations and extensions of the model are possible, building on the foundation established here, which combines aspects of both economic and econophysical approaches. Perhaps most obviously, the approximation of linearity could be relaxed because links between locations are often not bidirectional (e.g., the ability to load ore onto a ship does not necessarily imply the ability to unload it with equal ease at the same port) and have a maximum capacity at which their trade current will saturate or be subject to quotas; these effects can be simulated using nonlinear circuit elements such as diodes. Other nonlinear effects include economies of scale in production and the fact that supply and demand are also limited by maximum producer output, consumer budget constraints, and diminution of marginal utility at high volumes.

Other effects that could be included and explored in more detail would be holding of stockpiles and inventory in capacitive elements, wastage modeled via leak resistances, shocks via stepwise changes in supply, demand, and/or properties of trade links. Elaboration to include multiple, possibly competing, goods would be advantageous, as would the ability for producers to take traded commodities as inputs to more extensively transformed manufactured goods, which are then traded on their own markets. Reuse of waste via recycling could also be included by viewing it as a commodity, with negative price where necessary to subsidize recycling or pay for disposal. Inclusion of these effects in a future, extended analysis would require a model of consumer preference and utility as well as one of investment in capacity by producers seeking financial returns after allowing for the cost of capital. An investment model would also allow the evolution of transport and storage capacity by upgrading links and nodes in ways that are projected to generate a positive return on the required investment. More generally, all circuit parameters could be evolved, potentially to help to suppress excessive price fluctuations. Arbitrage could also be explored.
